# Vaccine Refusal in the Czech Republic Is Associated with Being Spiritual but Not Religiously Affiliated

**DOI:** 10.3390/vaccines9101157

**Published:** 2021-10-10

**Authors:** Alice Kosarkova, Klara Malinakova, Jitse P. van Dijk, Peter Tavel

**Affiliations:** 1 Olomouc University Social Health Institute, Palacky University in Olomouc, 77111 Olomouc, Czech Republic; klara.malinakova@oushi.upol.cz (K.M.); j.p.van.dijk@umcg.nl (J.P.v.D.); peter.tavel@oushi.upol.cz (P.T.); 2 Department of Community and Occupational Medicine, University Medical Center Groningen, University of Groningen, 9713GZ Groningen, The Netherlands; 3 Graduate School Kosice Institute for Society and Health, P. J. Safarik University in Kosice, 04011 Kosice, Slovakia

**Keywords:** religious conspiracy beliefs, COVID-19 vaccine, vaccination, spirituality, religiosity

## Abstract

A strong reduction in the deleterious effects of the COVID-19 pandemic can be achieved by vaccination. Religiosity and spirituality (R/S) may play an important role in vaccine acceptance. However, evidence is lacking for the associations with religious conspiracy theories (RCT) in a non-religious environment. This study investigated the associations between R/S and RCT about COVID-19 vaccination and the links of R/S with vaccine refusal and hesitancy. A sample of Czech adults (*n* = 459) participated in the survey. We measured R/S, RCT, religious fundamentalism, and COVID-19 vaccination intentions. We found spirituality to be significantly associated with RCT belief, with odds ratios (OR) of 2.12 (95% confidence interval [CI] 1.42–3.19). A combination of R/S groups revealed that spirituality with non-religious affiliation was associated with higher beliefs in RCT, with ORs from 3.51 to 7.17. Moreover, associations were found between spirituality with non-religious affiliation [OR 2.22(1.33–7.76)] with vaccine refusal. Our findings showed associations of spirituality and religious fundamentalism with RCT about COVID-19 vaccination. Furthermore, spirituality was linked to a higher possibility of vaccine refusal. Understanding these associations may help prevent the development of RCT and negative impact of spirituality on vaccine intentions and contribute to the effectiveness of the vaccination process.

## 1. Introduction

Vaccination is a means of health protection and plays a critical role in reducing the specific mortality rates of certain diseases [1,2]. In the current COVID-19 pandemic, millions of laboratory-confirmed cases of SARS-CoV-2 infection have been reported, along with 4 million reported deaths as of June 2021 [3]. Although there are ways to prevent the spread of infection, such as social distancing, contact tracing, testing, or the use of masks, these measures have been shown to be insufficient in reducing virus transmission and its consequences [4,5] when compared to vaccination. According to several studies, a significant reduction of COVID-19 morbidity and mortality can only be achieved by mass vaccination (e.g., [4,6,7]). Therefore, simultaneously with the spread of the virus, various COVID-19 vaccines have been developed by different pharmaceutical companies and subsequently approved by the European Medicines Agency (EMA). These are derived from multiple platforms and include different vaccines types [8,9,10].

However, no vaccine can reduce the pandemic without widespread acceptance [11], since the herd immunity resulting from vaccines may control or eliminate the infection only if the effective vaccination rate is sufficiently high [5,6] Moreover, low vaccination coverage may increase the emergence of more transmissible variants of the SARS-CoV-2 virus [3]. Yet, some individuals question, hesitate, or refuse particular vaccines or vaccination in general [12,13]. Similarly, studies on COVID-19 vaccination acceptance suggest that COVID-19 vaccine hesitancy and refusal are increasing worldwide on average [7,14]. Research has shown that the common reasons for COVID-19 vaccines refusal or hesitancy are a fear of side effects, safety and effectiveness, doubts about the correct development or approval of vaccines or their necessity, the unknown duration of immunity following vaccination, and a general anti-vaccine stance (see [14] for a review). Nevertheless, determinants of vaccine uptake can be more complex and multifaceted [14,15], involving cognitive, emotional, cultural, social, spiritual, or political factors [1; 13]. Moreover, the reasons contributing to hesitancy can be more specific to the particular individuals or subgroups within a population as well as the context [13]. Circumstances, such as attitudes, context, culture, and beliefs regarding COVID-19 vaccines, are therefore important factors in minimizing vaccination refusal or hesitancy.

Religiosity and spirituality (R/S) are factors that have been explored in relation to general vaccination attitudes [12,16,17,18], since they can empower some people to take responsibility for their health [19]. Religiosity, which can be described in terms of church attendance, institutional beliefs, and rituals and theology prescribed by a particular institution [20], has been explored as an important theme when discussing vaccination and shaping decisions for uptake [18,21,22]. Spirituality, perceived as an individual’s contentedness towards a Higher Power, a sense of the meaning of life, a search for harmony, and spiritual well-being [19], has been found to influence how people cope with health issues and care for their bodies [16,21]. However, spiritual worldviews [23] or moral issues connected to religion [24] can also be connected to general anti-vaccination behavior. Moreover, in all major religions, groups may be found with a strong adherence to their basic principles and a decisive expression of disagreement with modern society and science, which can be characterized as religious fundamentalism [25,26]. Fundamentalism has been associated with a devotion to strict religious interpretations and practices that provide clear rules for living, leaning towards dogma, distinctions between the secular and the religious, antimodernism, and to a sense that individuals’ lives are sanctioned and supported by God [27,28]. This ideology of religious exclusivity was found to be connected to general anti-vaccine attitudes [18,24] and attitudes towards COVID-19 vaccination [29,30]. Thus, it can represent a potential barrier to vaccine uptake [31] and be a source of hesitancy [1].

Moreover, social identity and the way people perceive the world around them can be associated with beliefs in conspiracy theories, particularly when events are unclear or uncertain [32,33]. Research suggests that beliefs in conspiracy theories (CT) have a negative influence on the health domain [32,34,35], including a harmful effect on vaccine uptake in general [36,37]. However, the COVID-19 pandemic has also mobilized groups spreading various CT [38,39,40], and such beliefs were found to be associated with distrust in COVID-19 vaccines [41], vaccine hesitancy, and refusal [42]. Although an individual’s R/S has been found to play a role in the endorsement of conspiracies, and a religious way of thinking may furthermore facilitate one’s attraction to conspiracies [32,43], studies on associations between R/S and RTC about COVID-19 vaccines are lacking. Therefore, it is important to understand the R/S foundations of worldviews that can support the formation of religious conspiracy theories (RCT) [30,44] and the possible links of R/S with COVID-19 vaccine intentions in order to strengthen this vaccine acceptance.

The Czech Republic, despite its Christian religious orientation [45], is characterized by a high degree of secularization, as most people do not report any religious affiliation or regular church attendance [45]. This setting can make it an interesting research area for assessing the links between R/S and beliefs in RCT and their associations with the COVID-19 vaccine uptake intentions. The findings from a secular country can help us to describe the basis from which beliefs in RCT may arise and to understand what variables may underlie decisions about vaccination. Therefore, the purpose of this study is to assess the associations between the role of R/S, religious fundamentalism, and beliefs in RCT and to examine their associations with the COVID-19 vaccine uptake.

## 2. Methods

### 2.1. Participants and Procedure

For this study, data from the Czech population aged 18 to 88 were obtained. The data were collected in April 2021 during the vaccination process, when nearly 10% of the Czech population was already fully vaccinated [46]. The online survey was prepared at the researcher’s institution and conducted by a professional agency Czech National Panel (ceskynarodnipanel.cz, accessed on 2 September 2021). This agency is one of the leading providers of online data collection and a reliable source of respondents for surveys in the Czech Republic. The participants were chosen with the help of quota sampling based on the criteria that allowed the construction of a sample close to the adult Czech representative samples. The agency used online methods of contacting the respondents, who are members of a stable panel and receive a reward for successful finishing of the questionnaire. This ensures achieving a balanced sample regarding age and gender. The number of respondents who received a prompt to join the survey is unknown, as it depended on repletion of individual quotas during the time. At the end of the data collection, the final sample was 1662 participants. However, visual screening indicated four cases of uniform pattern responses, i.e., responding to most of the items of the survey in the same way, which led to the exclusion of these respondents. After these four respondents were excluded, 1658 subjects remained. Consequently, in the next step, to ensure high quality of data, low-quality respondents were excluded following two criteria: (1) a very short period of time filling in the survey that would not have allowed responding to the questions thoughtfully (i.e., less than 15 min for a survey lasting around an hour); (2) responding inconsistently to control questions regarding years, weight, and height (i.e., respondents who reported a difference of two and more units of measure). After exclusion of these problematic subjects (*n* = 166), the remaining sample consisted of 1492 respondents. RCT beliefs and fundamentalism were assessed only among respondents who reported themselves as religious; thus, the final sample consisted of 459 participants (mean age = 51.46, SD = 16.05; 49.9% male).

At the beginning of the survey, participants received written information about the aim of the study and the anonymized handling of data and were made familiar with the system. Participation in the survey was fully voluntary; respondents had to explicitly express their informed consent with participation and had the possibility of leaving the study at any time without giving a reason. The study design was approved by the Ethics Committee of the Faculty of Theology, Palacký University in Olomouc (No. 2021/06).

### 2.2. Measures

Religiosity was measured using the following question: “At present, would you call yourself a believer?” with possible answers: “yes, I am a member of a church or religious society”; “yes, but I am not a member of a church or religious society”; “no”; “no, I am a convinced atheist”. For the purpose of our study, answering categories were dichotomized. At first, participants who chose the option “yes” were considered religious regardless of their proclaimed religious affiliation. Furthermore, participants who chose the option “yes, I am a member of a church or religious society” were considered religiously affiliated.

Spirituality was measured using the Daily Spiritual Experience Scale (DSES). The scale measures the frequency of ordinary experiences of connection with transcendence in everyday life [47]. The present study used an adapted 15-item version of the scale validated for the Czech environment [48]. The items are evaluated on a six-point modified Likert scale graded according to the intensity of the experience of the observed phenomena, ranging from “never” (1) to “many times a day” (6). The last item on the scale, the question “How close to God do you feel in general?” has only four options, ranging from “not at all” (1) to “as close as possible” (4). A higher intensity of experience corresponds to higher levels of spiritual experience. For the purposes of our analysis, the DSES score was treated as continuous, but for the assessment of different combinations of religious affiliation and spirituality with RCT, it was also dichotomized in the following way: We computed the total score ranging from 15 to 88 points. The respondents with a score of 51 or higher, i.e., above the middle of the score, were considered spiritual, and the rest as non-spiritual. Cronbach’s alpha for the whole scale has an excellent internal consistency, with α = 0.96 in our sample.

In order to distinguish between religious affiliation and spiritual experience and to assess their interaction, composite variables were created: (1) Spiritual and religiously affiliated; (2) Spiritual, but not religiously affiliated; (3) Non-spiritual, but religiously affiliated; and (4) Non-spiritual and not religiously affiliated.

COVID-19 vaccination intentions were assessed by a question: Will you be or have you already been vaccinated with a currently available COVID-19 vaccine? With possible answers: “no”, “I don’t know yet”, and “yes”. The response “no” was classified as vaccination refusal, the response “I don’t know yet” as vaccine hesitancy and “yes” as vaccine acceptance.

Religious conspiracy theories were assessed using statements capturing the common religious opinions on the COVID-19 vaccines. The statements were generated from searching the Internet and social media during the initial period of the vaccination against COVID-19 in 2021. Although the approach may not be completely exhaustive, we tried to capture the most common theories involving religious themes concerning COVID-19 vaccination. The assessed statements were e.g.: “Rejection of the COVID-19 vaccine is an act of true faith and trust in God.”; “The pope and false church prophets are fulfilling the intentions of world elites and spreading the ideas of modernism, which contradicts true tradition.”; “Some of the vaccines contain modified RNA that changes the human genome, which is a crime against the human race and its Creator”; “Vaccination is a sign of the end of the world”; “The current coronavirus pandemic is God’s punishment”; and “Vaccination with the COVID-19 vaccine is morally unacceptable because tissues from aborted foetuses were used for its development”. Participants were asked to mark to which degree, in their opinion, the information about COVID-19 vaccines or vaccination corresponds to the truth. Possible options ranged from “does not correspond at all” (0) to “definitely corresponds” (3). Consequently, when any of the four statements was marked as “corresponds” (2) or “definitely corresponds” (3), the respondent was classified as believing in the religious conspiracy theory.

Religious fundamentalism was measured using the Multi-Dimensional Fundamentalism Inventory (MDFI) [49]. The instrument was developed to assess a personal orientation that promotes a supra-human locus of moral authority, a contextual truth, and appreciation of the sacred over worldly experiences [49]. Therefore, the instrument comprises three subscales to measure three dimensions of religious fundamentalism: External versus internal authority; Fixed versus malleable religion; Rejection versus affirmation of the world. Each of the dimensions consists of five items rated on a 5-point Likert-type scale, ranging from “totally disagree” (1) to “totally agree” (5). For the purposes of our analysis, the MDFI score was treated as a continuous variable. Cronbach’s alpha for the MDFI total scale in the current sample was 0.62.

We obtained sociodemographic characteristics, such as gender, age, education level, marital status, and economic activity, from the questionnaire.

All instruments were available in the Czech language.

### 2.3. Statistical Analyses

As the first step, we described the background characteristics of the sample and attitudes towards vaccination and RCT beliefs. Non-parametric methods were used to compare different sociodemographic groups. The Wilcoxon signed-rank test was used to compare gender; in other cases, when more than two groups were compared, we used the Kruskal–Wallis test.

In the next step, we used binary logistic regression models, both crude and adjusted for gender, age, and education level. In a crude regression model, we assessed only one independent variable, i.e., spirituality, religious affiliation, and fundamentalism (each of the variables was assessed separately), with one dependent variable of interest, i.e., every single RCT, RCT sum, vaccine refusal, and vaccine hesitancy. The confounding variables in the adjusted model were age, gender, and education level. In Model 1, we assessed the associations of religious affiliation and spirituality with beliefs in RCT around COVID-19 vaccination (in total and each of the six theories separately). The different combinations of religious affiliation and spirituality with RCT were assessed in Model 2. Model 3 aimed to assess the associations of the MDFI with RCT. Subsequently, the multinominal logistic regression models were used to test the associations of R/S, their combinations, beliefs in RCT, and the MDFI with intentions towards COVID-19 vaccination. Each independent variable was tested in a separate model. Numeric variables were standardized to z-scores. All analyses were performed using the statistical software package IBM SPSS version 25 (IBM Corp., Armonk, NY, USA).

## 3. Results

### 3.1. Description of the Population

The sociodemographic characteristics of the sample are presented in Table 1. Of the whole sample (mean age = 51.46, SD = 16.05; 49.9% male), 24.6% of respondents hold RCT beliefs related to COVID-19 vaccination. Furthermore, 21.8% reported vaccine refusal and 22.2% vaccine hesitancy.

### 3.2. Beliefs in Religious Conspiracy Theories around COVID-19 Vaccination

Table 2 shows the associations of religious affiliation, spirituality, and their different combinations with the RCT related to COVID-19 vaccination. We assessed the following combinations of religious affiliation and spirituality: spiritual and religiously affiliated (S+RA; *n* = 55), spiritual but non-religiously affiliated (S+NRA; *n* = 35), non-spiritual but religiously affiliated (NS+RA; *n* = 72), non-spiritual and non-religiously affiliated (NS+NRA; *n* = 290). The number of respondents in each category is in line with the study of religiosity in the Czech Republic based on a representative sample [45] and other studies on R/S [50,51,52]. These results suggest that although some people from the Czech population consider themselves believers, they seeks spiritual fulfilment outside traditional religious institutions. We found that respondents with higher levels of spirituality were significantly more likely to believe in RCT, with odd ratios ranging from 1.37 (1.02–1.84) to 2.12 (1.42–3.19) for the adjusted model. Particularly, the strongest association was found for the opinion that the rejection of the COVID-19 vaccine is an act of true faith and trust in God. Moreover, a combination of groups revealed that spiritual but not religiously affiliated respondents had a significantly higher increase in the odds of RCT beliefs (ranging from 3.70 to 6.39).

### 3.3. COVID-19 Vaccine Intentions

On the contrary, the results indicated that religious affiliation was not associated with beliefs in RCT. In addition, being non-spiritual but religiously affiliated was significantly associated with a lower probability of RCT beliefs (a 52% decrease in the odds).

Furthermore, higher levels of religious fundamentalism were associated with some of the assessed RTC, with the most significantly associated belief that the current coronavirus pandemic is God’s punishment (an 89% increase in the odds).

Table 3 depicts the results of multinominal logistic regression, with the cluster of respondents who accepted vaccination as the reference category. Attitudes towards vaccination were assessed in association with RCT beliefs, R/S and their different combinations, and with fundamentalism. Spiritual respondents were more likely (a 37% increase in the odds) to refuse vaccination. Moreover, compared to non-spiritual non-affiliated respondents, respondents who were spiritual but non-religiously affiliated were about 4.43 times more likely to refuse the vaccination. Similarly, this group had a significantly (2.88 times) higher chance to hesitate regarding vaccine acceptance.

## 4. Discussion

The aim of this study was to assess the relationship of R/S and religious fundamentalism with beliefs in RCT about COVID-19 vaccination, as well as to explore the links of R/S with attitudes towards vaccination in the Czech Republic. We found that higher levels of spirituality and of fundamentalism were associated with beliefs in RCT around the COVID-19 vaccination. The associations of spirituality were even stronger when spirituality was combined with non-affiliation with a religious organization, whereas members affiliated with religious organizations did not report RCT beliefs. Moreover, we found that spirituality, both itself and in combination with non-affiliation, was associated with increased levels of vaccine refusal. RCT beliefs were also significantly linked to vaccine refusal.

We found strong associations with RCT beliefs with spirituality, whereas religious affiliation was not found to be associated with such beliefs. In distinguishing religiosity and spirituality as two different concepts [20,50], we may suppose that individuals with higher levels of spirituality, in their efforts to find and explain the meaning of COVID-19, lean towards conspiracy ideas connected to their spiritual worldviews [33,53]. Thus, the associations of spirituality with the assessed RCT beliefs are in line with studies that showed that personal ideology and individual attitudes, including esotericism and belief in the healing and sacred power of one’s own body, play a fundamental role in the creation of and beliefs in conspiracy theories [53]. Similarly, beliefs in RCT can reflect perceived threats and assimilate spiritual thoughts into the narrative structure in which they exist [30,54]. They may also stem from seeking spiritual purity, an effort to create an ideal reinterpreted past or cling to a perfect post-apocalyptic era [30,55]. Therefore, respondents with higher levels of spirituality may have a tendency to believe in RCT that are based on apocalyptic ideas or defend alternative forms of medicine [23].

Our findings of no associations between beliefs in RCT and religiosity are in contrast to studies of Marchlewska et al. [56] or Sturm and Albrecht [29]. These studies were conducted in countries with the predominant Christian religion, such as Poland [56] or the USA [29], nevertheless, their research focus was either not connected to vaccination but focused on the foundations of the Christian faith and morals, i.e., RCT about gender and marriage [56], or used narratives specific to Christian narratives, i.e., apocalyptical or millennial [29]. Thus, we can suppose that the affiliated respondents did not see the RCT beliefs around COVID-19 vaccination as threatening their religious identity, interfering with the teachings of the church [39,56], or as a sign of the end of the world. Moreover, as our study was conducted after the Catholic church released an official encouragement for people to get vaccinated [57], we may assume that our respondents, whose denomination is mainly Catholic, were following the teaching of the church and did not link COVID-19 vaccines with RCT. Nevertheless, beliefs in RCT were found to be associated with fundamentalism. Therefore, we may assume that not the affiliation to religion itself but the specific type of religious involvement may play an essential role in RCT endorsement. These results are in line with the findings of some other authors [32,58], who showed links between identification with a specific religious ingroup and reinforced beliefs in conspiracy theories and with perceiving non-religious outgroups as immoral and evil. Religious fundamentalism with conservative views and clinging to traditions and anti-modernism [55] may lead to perceiving outgroups as underestimating moral values that their religion represents [30,56] and threatens the ingroup identity [32]. Moreover, the type of religion may be connected to the way some people use their R/S to cope with and understand difficult life situations [59]. They may use strategies characterized by an ominous view of the world and conflict with people in a religious community. Therefore, not religion itself, but strong attachment to its specific forms may reinforce beliefs in RCT and increase their demarcation from others, even within the same religion [39,53]. In addition, these extreme believers can spread RCT very effectively, because they often have a social network in which such theories can be mutually supported by like-minded people [23].

In our study, we found that spiritual respondents reported refusal and hesitancy regarding COVID-19 vaccination. Similarly, the combination of groups revealed that spirituality without being religiously affiliated was linked to high levels of vaccination refusal and hesitancy, whereas affiliation to a church showed no significant associations. These results are in line with studies showing that spiritual attitudes may be among the reasons for vaccine refusal [16,21,60]. The factors associated with spiritual objections may comprise a belief in the natural healing potential of the body and in alternative forms of medicine, including prayer and strong faith [23,60], moral issues regarding the content of a vaccine, or the conviction that the disease is given by a Higher Power and can be withstood by the immune system [61]. However, the findings of our study showed a discrepancy with authors that identified religion as a barrier to vaccine uptake and a source of hesitancy [1,18,31]. The majority of religions do not have doctrinal objections to vaccination, and vaccines are treated as an important measure to preserve health, “to care for the temple of one’s body”, and to strengthen solidarity with others through the protection of the entire society [12,24,62]. This is why we may argue that not religion itself but only some religious communities, usually orthodox or with conservative interpretations of scripture, may share negative attitudes towards vaccination [18,51,63], as shown in our study conducted in a secular country where only a low percentage of religious people are predominantly Christian. Therefore, our results based on data from the secular environment of the Czech Republic are rather in line with studies, showing that spirituality without religious affiliation may lead to health-risk behavior [52,64], supporting this idea even in the field of vaccination.

Our study indicates that spirituality without being religiously affiliated is significantly related to RCT beliefs around COVID-19 vaccines, suggesting that people who are spiritual but not affiliated are more likely refuse the COVID-19 vaccine.

### 4.1. Strengths and Limitations

This study has some important strengths. First, it is one of the few studies exploring the relationship between RCT about COVID-19 vaccination, R/S areas of human life, and vaccination intentions, and describing significant associations in this area. Further, with its focus on a specific area of religious conspiracies, it contributes to other studies that found possible links between COVID-19 conspiracy beliefs with vaccine refusal or hesitancy.

However, this study also has some limitations. The first is the cross-sectional design, which does not enable us to make decisive conclusions on the direction of causality. Thus, the present study should be confirmed by studies with a longitudinal design. Another limitation can be that due to the small sample of religious respondents, we were not able to assess different religious communities and church denominations. We are aware of the fact that this could have led to more specific study results. Nevertheless, our study comes with findings on religiosity in a general way. A further limitation can be that our measures may not have captured all relevant RCT known to the sample. However, having searched various social media, we tried to encompass and formulate the most common and shared ones. In addition, our study used a self-report methodology, which can cause information bias and may be influenced by a social desirability. Nevertheless, in the area of assessing conspiracy theory beliefs, an online anonymous survey seems to be an applicable means of lowering the unwillingness of respondents to admit their true beliefs [65].

### 4.2. Implications

Our results show that RCT beliefs concerning COVID-19 vaccination are related to an individual’s spirituality and to being spiritual but not religiously affiliated. These findings may help to understand factors that influence the dynamics of RCT development and their associations with R/S areas. We also found that both spirituality and RCT were positively associated with refusal of a COVID-19 vaccine. This indicates that some aspects of R/S may have a relevant impact on the development and spreading of conspiracy theories as well as on taking a decision on vaccination. This information may be helpful for health care workers, as well as for workers in helping professions, such as psychotherapy or pastoral care. Moreover, it can be informative and useful for all those working on vaccination campaigns to prevent the spread of the coronavirus pandemic and help them choose appropriate strategies to also reach this subgroup of inhabitants.

Further research should focus on the causal effects of the RCT beliefs dynamic and on the mutual interaction between R/S and conspiracy theories in general. It could also focus on the more specific reasons for vaccine refusal apart from conspiracies and test for potential confounders between R/S, RCT, and vaccine intentions.

## 5. Conclusions

Vaccination against COVID-19 reduces its detrimental effects on human health and society. However, this requires widespread acceptance of the majority of the population. Our findings emphasize the associations of R/S and religious fundamentalism about COVID-19 vaccine with spirituality and religious fundamentalism in the Czech Republic. A negative effect was further revealed by significantly higher levels of COVID-19 vaccine refusal among those who were spiritual but not religiously affiliated. Thus, this study offers a deeper understanding of the factors that might influence the development of religious conspiracy theories and the extent to which these beliefs may affect vaccine intentions. Furthermore, it stresses the importance of addressing spiritual issues in order to minimize vaccine refusal associated with being spiritual but not religiously affiliated.

## Figures and Tables

**Table 1:** Description of the study sample.

	Total		COVID-19 Vaccine Intentions						Beliefs in RCT ^1^		
			Refusal			Hesitancy					
	N	%	N	%	*p*-value	N	%	*p*-value	N	%	*p*-value
Sex											
Male	229	49.9	45	45.0	n.s.	51	50.0	n.s.	55	48.7	n.s
Female	230	50.1	55	55.0		51	50.0		58	51.3	
Age											
18–34	81	17.6	22	22.0	0.001	23	22.5	0.015	15	13.3	n.s.
35–49	150	32.7	44	44.0	(1–4 *)	40	39.2	(1–4 *)	36	31.9	
50–65	106	23.1	21	21.0	(2–4 **)	24	23.5	(2–4 *)	30	26.5	
66–99	122	26.6	13	13.0		15	14.7		32	28.3	
Marital status											
Married/partnership	288	62.7	57	57.0	n.s.	59	57.8	n.s.	70	61.9	n.s.
Single/divorced/widow(er)	171	37.3	43	43.0		43	42.2		43	38.1	
Economic status											
Student	14	3.1	2	2.0	0.003	6	5.9	n.s.	1	0.9	n.s.
Employee	202	44.0	47	47.0	(4–5 *)	48	47.1		44	38.9	
Self-employed	30	6.5	11	11.0		3	2.9		8	7.1	
Disabled/old-age pensioner	173	37.7	25	25.0		33	32.4		49	43.4	
Household ^2^/unemployed	40	8.7	15	15.0		12	11.8		11	9.7	
Education level											
Elementary	31	6.8	9	9.0	n.s.	5	4.9	n.s.	8	7.1	n.s.
Secondaryvocational	174	37.9	46	46.0		48	47.1		52	46.0	
Secondary graduation	130	28.3	22	22.0		30	29.4		36	31.9	
College	124	27.0	23	23.0		19	18.6		17	15.0	
Affiliation											
Member of a church	131	28.5	23	23.0	n.s.	28	27.5	n.s.	26	23.0	n.s.
Non-affiliated	328	71.5	77	77.0		74	72.5		87	77.0	
Total	459	100.0	100	21.8		102	22.2		113	24.6	

Notes: ^1^ believing in at least one religious conspiracy theory; ^2^ including maternity leave; n.s. = non-significant. * *p* < 0.05, ** *p* < 0.01. The *p*-value stands for comparison of all groups; results in parentheses show multiple-group comparison with Bonferroni correction.

**Table 2:** Associations of religious affiliation, spirituality (standardised to Z-scores), different combinations of religious affiliation and spirituality, and religious fundamentalism (standardised to Z-scores) with RCT beliefs: Results of binary logistic regression (crude and adjusted) for age, gender, and education level leading to odds ratios (OR) with 95% confidence intervals.

		RCT1	RCT2	RCT3	RCT4	RCT5	RCT6	RCT Sum
Model 1								
Religious affiliation	Crude ^1^	0.67 (0.22–2.07)	0.44 (0.16–1.15)	0.67 (0.22–2.07)	1.00 (0.54–1.85)	1.12 (0.57–2.23)	0.47 (0.20–1.09)	0.69 (0.42–1.13)
	Adjusted ^2^	0.74(0.24–2.32)	0.44 (1.17–1.19)	0.80 (0.26–2.50)	1.13 (0.60–2.14)	1.25 (0.62–2.53)	0.46 (0.20–1.08)	0.74 (0.44–1.22)
Spirituality	Crude ^1^	2.05 (1.38–3.0) ***	1.42(1.03–1.95) *	1.60 (1.07–2.40) *	1.30 (1.00–1.69) *	1.26 (0.94–1.69)	1.34(1.00–1.79) *	1.33 (1.09–1.64) **
	Adjusted ^2^	2.12 (1.42–3.19) ***	1.49 (1.08–2.06) *	1.62 (1.08–2.43) *	1.37 (1.05–1.78) *	1.26 (0.94–1.70)	1.37 (1.02–1.84) *	1.38 (1.12–1.70) **
Model 2								
S+RA	Crude ^1^	2.45 (0.73–8.25)	0.78 (0.22–2.71)	1.62 (0.43–6.07)	1.62 (0.75–3.50)	1.55 (0.63–3.78)	0.58 (0.17–1.98)	1.22 (0.65–2.31)
	Adjusted ^2^	2.79 (0.81–9.64)	0.85 (0.24–3.02)	1.81 (0.47–6.94)	1.84 (0.83–4.08)	1.65 (0.66–4.12)	0.57 (0.17–1.99)	1.32 (0.68–2.53)
S+NRA	crude ^1^	6.46 (2.15–19.44) **	4.00 (1.61–9.94) **	4.50 (1.50–14.56) **	1.51 (0.58–3.89)	1.77 (0.63–4.96)	3.54 (1.50–8.36) **	1.29 (1.12–4.71) *
	Adjusted ^2^	7.17 (2.27–22.67) **	4.64 (1.79–12.03) ***	4.56 (1.40–14.84) **	1.56 (0.59–4.14)	1.75 (0.61–5.07)	3.51 (1.45–8.50) **	2.34 (1.11–4.92) *
NS+RA	crude ^1^	^a^	0.39 (0.08–1.69)	0.39 (0.05–3.13)	0.66 (0.27–1.64)	0.96 (0.38–2.45)	0.56 (0.19–1.66)	0.49 (0.24–1.00) *
	Adjusted ^2^	^a^	0.37 (0.08–1.66)	0.49 (0.06–3.98)	0.74 (0.29–1.87)	1.16 (0.44–3.01)	0.55 (0.18–1.66)	0.51 (0.24–1.06) *
NS+NRA		1	1	1	1	1	1	1
Model 3								
Fundamentalism	Crude ^1^	1.86 (1.17–2.98) **	1.71(0.79–3.73)	1.61 (1.01–2.57) *	1.21 (0.91–1.61)	1.88 (1.35–2.61) ***	0.94 (0.68–1.29)	1.31 (0.05–1.62) *
	Adjusted ^2^	1.83 (1.13—2.96) *	1.26 (0.88—1.81)	1.53 (0.95–2.45)	1.18 (0.88–1.57)	1.89 (1.34–2.68) ***	0.92 (0.66–1.27)	1.27 (1.02–1.59) *

Notes: * *p* < 0.05, ** *p* < 0.01, *** *p* < 0.001. ^1^ a crude independent variable assessed with a dependent variable. ^2^ an independent variable together with age, gender, and education level assessed with a dependent variable. **RCT1**–rejection of the COVID–19 vaccine is an act of true faith and trust in God; **RCT2**—the vaccine contains modified RNA that changes the human genome, which is a crime against the human race and its Creator; **RCT3**—vaccination is a sign of the end of the world; **RCT4**—the pope and false church prophets are fulfilling the intentions of world elites and spreading the ideas of modernism, which contradicts true tradition; **RCT5**—the current coronavirus pandemic is God’s punishment; **RCT6**—vaccination with the COVID-19 vaccine is morally unacceptable, because tissues from aborted foetuses were used for its development. **S+RA** = spiritual and religiously affiliated; **S+NRA** = spiritual but non-religiously affiliated; **NS+RA** = non-spiritual but religiously affiliated; **NS+NRA** = non-spiritual and non-religiously affiliated **^a^** RCT1 (S+RA) was not possible to estimate due to the low number of respondents in this category; the regression model did not converge.

**Table 3:** Associations of religious affiliation, spirituality (standardised to Z-scores), different combinations of religious affiliation and spirituality, and religious fundamentalism (standardised to Z-scores) with attitudes towards COVID-19 vaccination: results of multinominal logistic regression crude and adjusted for age, gender, and education level, leading to odds ratios (OR) with 95% confidence intervals.

		Vaccine Refusal	Vaccine Hesitancy
Model 1			
Non-affiliated vs. affiliated	Crude ^1^	0.66 (0.39–1.13)	0.84 (0.50–1.39)
	Adjusted ^2^	0.79 (0.45–1.39)	1.03 (0.60–1.78)
Spirituality	Crude ^1^	1.35 (1.08–1.69) **	0.08 (0.85–1.37)
	adjusted ^2^	1.37 (1.08–1.73) **	1.12 (0.87–1.44)
Model 2			
S+RA	Crude ^1^	1.08 (0.53–2.18)	0.99 (0.48–2.05)
	Adjusted ^2^	1.20 (0.57–2.53)	1.18 (0.55–2.58)
S+NRA	Crude ^1^	2.78 (1.20–6.41) **	2.14 (0.88–5.19)
	Adjusted ^2^	2.22 (1.33–7.76) **	2.74 (1.07–7.00) *
NS+RA	Crude ^1^	0.53 (0.26–1.12)	0.80 (0.42–1.53)
	Adjusted ^2^	0.77 (0.32–1.51)	1.06 (0.60–2.11)
NS + NRA		1	1
Model 3			
MDFI	Crude ^1^	1.21 (0.95–1.52)	1.16 (0.91–1.46)
	Adjusted ^2^	1.16 (0.91–1.48)	1.12 (0.88–1.43)

Notes: * *p* < 0.05, ** *p* < 0.01, *** *p* < 0.001. ^1^ a crude independent variable assessed with a dependent variable. ^2.^ an independent together with age, gender, and education level assessed with a dependent variable. S+RA = spiritual and religiously affiliated; S+NRA = spiritual but non-religiously affiliated; NS+RA = non-spiritual but religiously affiliated; NS+NRA = non-spiritual and non-religiously affiliated. MDFI = Multi-Dimensional Fundamentalism Inventory; RCT = religious conspiracy theories.

## Data Availability

The data for this article will be shared on reasonable request to the corresponding author.

## References

[1] Dubé E.D., Gagnon D., Macdonald N.E. (2015). Strategies intended to address vaccine hesitancy: Review of published reviews. Vaccine.

[2] Harrison E.A., Wu J.W. (2020). Vaccine confidence in the time of COVID-19. Eur. J. Epidemiol..

[3] World Health Organization COVID-19 Weekly Epidemiological Update 49. 20 July 2021–26 July 2021. https://www.who.int/emergencies/diseases/novel-coronavirus-2019/situation-reports.

[4] Baden L.R., El Sahly H.M., Essink B., Kotloff K., Frey S., Novak R., Diemert D., Spector S.A., Rouphael N., Creech C.B. (2021). Efficacy and Safety of the mRNA-1273 SARS-CoV-2 Vaccine. N. Engl. J. Med..

[5] Hogan C.A., Sahoo M.K., Pinsky B.A. (2020). Sample Pooling as a Strategy to Detect Community Transmission of SARS-CoV-2. JAMA.

[6] Saad-Roy C.M., Wagner C.E., Baker R.E., Morris S.E., Farrar J., Graham A.L., Levin S.A., Mina M.J., Metcalf C.J.E., Grenfell B.T. (2020). Immune life history, vaccination, and the dynamics of SARS-CoV-2 over the next 5 years. Science.

[7] Schwarzinger M., Watson V., Arwidson P., Alla F., Luchini S. (2021). COVID-19 vaccine hesitancy in a representative working-age population in France: A survey experiment based on vaccine characteristics. Lancet Public Health.

[8] Walsh E.E., Frenck R.W., Falsey A.R., Kitchin N., Absalon J., Gurtman A., Lockhart S., Neuzil K., Mulligan M.J., Bailey R. (2020). Safety and Immunogenicity of Two RNA-Based COVID-19 Vaccine Candidates. N. Engl. J. Med..

[9] Krammer F. (2020). SARS-CoV-2 vaccines in development. Nature.

[10] Zhang Y., Zeng G., Pan H., Li C., Hu Y., Chu K., Han W., Chen Z., Tang R., Yin W. (2021). Safety, tolerability, and immunogenicity of an inactivated SARS-CoV-2 vaccine in healthy adults aged 18–59 years: A randomised, double-blind, placebo-controlled, phase 1/2 clinical trial. Lancet Infect. Dis..

[11] Skjefte M., Ngirbabul M., Akeju O., Escudero D., Hernandez-Diaz S., Wyszynski D.F., Wu J.W. (2021). COVID-19 vaccine acceptance among pregnant women and mothers of young children: Results of a survey in 16 countries. Eur. J. Epidemiol..

[12] Grabenstein J.D. (2013). What the world’s religions teach, applied to vaccines and immune globulins. Vaccine.

[13] Larson H.J., Jarrett C., Schulz W.S., Chaudhuri M., Zhou Y., Dube E., Schuster M., MacDonald N.E., Wilson R., SAGE Working Group on Vaccine Hesitancy (2015). Measuring vaccine hesitancy: The development of a survey tool. Vaccine.

[14] Lin C., Tu P., Beitsch L.M. (2020). Confidence and Receptivity for COVID-19 Vaccines: A Rapid Systematic Review. Vaccines.

[15] Yin F., Wu Z., Xia X., Ji M., Wang Y., Hu Z. (2021). Unfolding the Determinants of COVID-19 Vaccine Acceptance in China. J. Med. Internet Res..

[16] Best A.L., Thompson E.L., Adamu A.M., Logan R., Delva J., Thomas M., Cunningham E., Vamos C., Daley E. (2019). Examining the Influence of Religious and Spiritual Beliefs on HPV Vaccine Uptake Among College Women. J. Relig. Health.

[17] Larson H.J., De Figueiredo A., Xiahong Z., Schulz W.S., Verger P., Johnston I.G., Cook A.R., Jones N.S. (2016). The State of Vaccine Confidence 2016: Global Insights Through a 67-Country Survey. EBioMedicine.

[18] Ruijs W.L.M., Hautvast J.L.A., Van Ijzendoorn G., Van Ansem W.J.C., Van Der Velden K., Hulscher M.E. (2012). How orthodox protestant parents decide on the vaccination of their children: A qualitative study. BMC Public Health.

[19] Koenig H.G. (2012). Religion, Spirituality, and Health: The Research and Clinical Implications. ISRN Psychiatr..

[20] Zinnbauer B.J., Pargament K.I., Cole B., Rye M.S., Butter E.M., Belavich T.G., Hipp K.M., Scott A.B., Kadar J.L. (1997). Religion and Spirituality: Unfuzzying the Fuzzy. J. Sci. Study Relig..

[21] Thomas T., Blumling A., Delaney A. (2015). The Influence of Religiosity and Spirituality on Rural Parents’ Health Decision Making and Human Papillomavirus Vaccine Choices. Adv. Nurs. Sci..

[22] Shelton R.C., Snavely A.C., De Jesus M., Othus M.D., Allen J.D. (2013). HPV Vaccine Decision-Making and Acceptance: Does Religion Play a Role?. J. Relig. Health.

[23] Larson H., Fleck F. (2014). Underlying issues are key to dispelling vaccine doubts. Bull. World Health Org..

[24] Pelcic G., Karacic S., Mikirtichan G.L., Kubars O.I., Leavitt F., Cheng-TekTai M., Morishita N., Vuletic S., Tonnasevic L. (2016). Religious exception for vaccination or religious excuses for avoiding vaccination. Croat. Med. J..

[25] Nagata J. (2001). Beyond Theology: Toward an Anthropology of “Fundamentalism”. Am. Anthropol..

[26] Altemeyer B., Hunsberger B. (1992). Authoritarianism, religious fundamentalism, quest, and prejudice. Int. J. Psychol. Relig..

[27] Altemeyer B., Hunsberger B. (2004). A Revised Religious Fundamentalism Scale: The Short and Sweet of it. Int. J. Psychol. Relig..

[28] Pargament K.I. (2002). The bitter and the sweet: An evaluation of the costs and benefits of religiousness. Psychol. Inq..

[29] Whitehead A.L., Perry S.L. (2020). How Culture Wars Delay Herd Immunity: Christian Nationalism and Anti-vaccine Attitudes. Socius Soc. Res. Dyn. World.

[30] Sturm T., Albrecht T. (2021). Constituent COVID-19 apocalypses: Contagious conspiracism, 5G, and viral vaccinations. Anthropol. Med..

[31] Costa J.C., Weber A.M., Darmstadt G.L., Abdalla S., Victora C.G. (2020). Religious affiliation and immunization coverage in 15 countries in Sub-Saharan Africa. Vaccine.

[32] van Prooijen J.W., Douglas K.M. (2018). Belief in conspiracy theories: Basic principles of an emerging research domain. Eur. J. Soc. Psychol..

[33] Douglas K.M., Sutton R.M., Callan M.J., Dawtry R.J., Harvey A.J. (2016). Someone is pulling the strings: Hypersensitive agency detection and belief in conspiracy theories. Think. Reason..

[34] Abaido G.M., Takshe A.A. (2020). COVID-19: Virus or Viral Conspiracy Theories?. Am. J. Biomed. Sci..

[35] Imhoff R., Lamberty P. (2018). How paranoid are conspiracy believers? Toward a more fine-grained understanding of the connect and disconnect between paranoia and belief in conspiracy theories. Eur. J. Soc. Psychol..

[36] Jolley D., Douglas K.M. (2014). The Effects of Anti-Vaccine Conspiracy Theories on Vaccination Intentions. PLoS ONE.

[37] Douglas K.M., Sutton R.M., Jolley D., Wood M.J., Bilewicz M., Cichocka A., Soral W.W. (2015). The social, political, environmental, and health-related consequences of conspiracy theories: Problems and potential solutions. The Psychology of Conspiracy.

[38] Sallam M., Dababseh D., Yaseen A., Al-Haidar A., Ababneh N.A., Bakri F.G., Mahafzah A. (2020). Conspiracy Beliefs Are Associated with Lower Knowledge and Higher Anxiety Levels Regarding COVID-19 among Students at the University of Jordan. Int. J. Environ. Res. Public Health.

[39] Kim S., Kim S. (2020). Searching for General Model of Conspiracy Theories and Its Implication for Public Health Policy: Analysis of the Impacts of Political, Psychological, Structural Factors on Conspiracy Beliefs about the COVID-19 Pandemic. Int. J. Environ. Res. Public Health.

[40] Allington D., Duffy B., Wessely S., Dhavan N., Rubin J. (2020). Health-protective behaviour, social media usage and conspiracy belief during the COVID-19 public health emergency. Psychol. Med..

[41] Bertin P., Nera K., Delouvee S. (2020). Conspiracy Beliefs, Rejection of Vaccination, and Support for hydroxychloroquine: A Conceptual Replication-Extension in the COVID-19 Pandemic Context. Front. Psychol..

[42] Sallam M., Dababseh D., Eid H., Al-Mahzoum K., Al-Haidar A., Taim D., Yaseen A., Ababneh N.A., Bakri F.G., Mahafzah A. (2021). High Rates of COVID-19 Vaccine Hesitancy and Its Association with Conspiracy Beliefs: A Study in Jordan and Kuwait among Other Arab Countries. Vaccines.

[43] Beller J. (2017). Religion and Militarism: The Effects of Religiosity, Religious Fundamentalism, Religious Conspiracy Belief, and Demographics on Support for Military Action. Peace Confl..

[44] Bezalel G.Y. (2019). Conspiracy Theories and Religion: Reframing Conspiracy Theories as Bliks. Episteme.

[45] Furstova J., Malinakova K., Sigmundova D., Tavel P. (2021). Czech Out the Atheists: A Representative Study of Religiosity in the Czech Republic. Int. J. Psychol. Relig..

[46] Ministry of Health of the Czech Republic (2021). Aktualne o koronaviru—COVID-19 Epidemic. https://koronavirus.mzcr.cz/.

[47] Underwood L.G. (2006). Ordinary Spiritual Experience: Qualitative Research, Interpretive Guidelines, and Population Distribution for the Daily Spiritual Experience Scale. Arch. Psychol. Relig..

[48] Malinakova K., Trnka R., Sarnikova G., Smekal V., Furstova J., Tavel P. (2018). Psychometric evaluation of the Daily Spiritual Experience Scale (DSES) in the Czech environment. Czech. Psychol..

[49] Liht J., Conway L.G.I., Savage S., White W., O’Neill K.A. (2011). Religious fundamentalism: An empirically derived construct and measurement scale. Arch. Psychol. Relig..

[50] Kosarkova A., Malinakova K., Koncalova Z., Tavel P., van Dijk J.P. (2020). Childhood Trauma Is Associated with the Spirituality of Non-Religious Respondents. Int. J. Environ. Res. Public Health.

[51] McDuffie D.C. (2020). Sacred immunity: Religion, vaccines, and the protection of public health in America. J. Public Health.

[52] Buchtova M., Malinakova K., Kosarkova A., Husek V., Van Dijk J.P., Tavel P. (2020). Religious Attendance in a Secular Country Protects Adolescents from Health-Risk Behavior Only in Combination with Participation in Church Activities. Int. J. Environ. Res. Public Health.

[53] Robertson D.G., Asprem E., Dyrendal A., Robertson D.G., Asprem E., Dyrendal A. (2018). Introducing the Field: Conspiracy Theory in, about, and as Religion. Handbook of Conspiracy Theory and Contemporary Religion.

[54] Howard R.G. (2006). Sustainability and Narrative Plasticity in Online Apocalyptic Discourse after September 11, 2001. J. Media Relig..

[55] Savage S., Liht J. (2008). Mapping fundamentalisms: The psychology of religion as a sub discipline in the prevention of religiously motivated violence. Arch. Psychol. Relig..

[56] Marchlewska M., Cichocka A., Łozowski F., Górska P., Winiewski M. (2019). In search of an imaginary enemy: Catholic collective narcissism and the endorsement of gender conspiracy beliefs. J. Soc. Psychol..

[57] Vatican COVID-19 Commission in collaboration with the Pontifical Academy for Life (2020). Vaccine for all. 20 Points for a Fairer and Healthier World. https://press.vatican.va/content/salastampa/it/bollettino/pubblico/2020/12/29/0697/01628.html#notaing.

[58] Cichocka A. (2016). Understanding defensive and secure in-group positivity: The role of collective narcissism. Eur. Rev. Soc. Psychol..

[59] Pargament K., Feuille M., Burdzy D. (2011). The Brief RCOPE: Current Psychometric Status of a Short Measure of Religious Coping. Religions.

[60] Browne M., Thomson P., Rockloff M.J., Pennycook G. (2015). Going Against the herd: Psychological and cultural factors Underly-ing the Vaccination confidence Gap. PLoS ONE.

[61] Rumetta J., Abdul-Hadi H., Lee Y.K. (2020). A qualitative study on parents’ reasons and recommendations for childhood vaccination refusal in Malaysia. J. Infect. Public Health.

[62] (2019). Pontifical Academy for Life Statement: Moral Reflections on Vaccines Prepared from Cells Derived from Aborted Human Foetuses. Linacre Q..

[63] Lisowski B., Yuvan S., Bier M. (2019). Outbreaks of the measles in the Dutch Bible Belt and in other places—New prospects for a 1000 year old virus. Biosystems.

[64] Malinakova K., Geckova A.M., van Dijk J.P., Kalman M., Tavel P., Reijneveld S.A. (2018). Adolescent religious attendance and spirituality-Are they associated with leisure-time choices?. PLoS ONE.

[65] Wood M.J., Douglas K.M. (2015). Online communication as a window to conspiracist worldviews. Front. Psychol..

